# BLEND-A: blending internet treatment into conventional face-to-face treatment for alcohol use disorder - a study protocol

**DOI:** 10.1186/s12888-021-03122-4

**Published:** 2021-03-06

**Authors:** Angelina Isabella Mellentin, Silke Behrendt, Randi Bilberg, Matthijs Blankers, Marie Paldam Folker, Kristine Tarp, Jakob Uffelmann, Anette Søgaard Nielsen

**Affiliations:** 1 Department of Clinical Research, Unit of Clinical Alcohol Research, University of Southern Denmark, Odense, Denmark; 2 Psychiatric University Hospital, University Function, Region of Southern Denmark Odense, Denmark; 3 Research Unit for Telepsychiatry and E-mental Health, Centre for Telepsychiatry in the Mental Health Services in the Region of Southern Denmark and Department of Clinical Research, University of Southern Denmark, Odense, Denmark; 4 Department of Clinical Research, I BRIDGE, Brain Research, Inter-Disciplinary Guided Excellence, University of Southern Denmark, Odense, Denmark; 5 Institute for Psychology, University of Southern Denmark, Odense, Denmark; 6 Department of Research, Arkin Mental Health Care, Amsterdam, The Netherlands; 7 Department of Psychiatry, Amsterdam UMC, location AMC, University of Amsterdam, Amsterdam, The Netherlands; 8 Trimbos Institute – The Netherlands Institute of Mental Health and Addiction, Utrecht, The Netherlands; 9 Sundhed.dk, Copenhagen, Denmark; 10 OPEN, Open Patient data Explorative Network, Odense University Hospital, Odense, Denmark

**Keywords:** Alcohol use disorder, Blended treatment, Cognitive behavior therapy, Guided internet-based treatment, Motivational interviewing

## Abstract

**Background:**

A major challenge to psychological treatment for alcohol use disorder (AUD) is patient non-compliance. A promising new treatment approach that is hypothesized to increase patient compliance is blended treatment, consisting of face-to-face contact with a therapist combined with modules delivered over the internet within the same protocol. While this treatment concept has been developed and proven effective for a variety of mental disorders, it has not yet been examined for AUD.

**Aims:**

The study described in this protocol aims to examine and evaluate patient compliance with blended AUD treatment as well as the clinical and cost effectiveness of such treatment compared to face-to-face treatment only.

**Methods:**

The study design is a pragmatic, stepped-wedge cluster randomized controlled trial. The included outpatient institutions (planned number of patients: *n* = 1800) will be randomized in clusters to implement either blended AUD treatment or face-to-face treatment only, i.e. treatment as usual (TAU). Both treatment approaches consist of motivational interviewing and cognitive behavioral therapy. Data on sociodemographics, treatment (e.g. intensity, duration), type of treatment conclusion (compliance vs. dropout), alcohol consumption, addiction severity, consequences of drinking, and quality of life, will be collected at treatment entry, at treatment conclusion, and 6 months after treatment conclusion. The primary outcome is compliance at treatment conclusion, and the secondary outcomes include alcohol consumption and quality of life at six-months follow-up. Data will be analyzed with an Intention-to-treat approach by means of generalized linear mixed models with a random effect for cluster and fixed effect for each step. Also, analyses evaluating cost-effectiveness will be conducted.

**Discussion:**

Blended treatment may increase treatment compliance and thus improve treatment outcomes due to increased flexibility of the treatment course. Since this study is conducted within an implementation framework it can easily be scaled up, and when successful, blended treatment has the potential to become an alternative offer in many outpatient clinics nationwide and internationally.

**Trial registration:**

Clinicaltrials.gov.: NCT04535258, retrospectively registered 01.09.20.

## Background

There is general agreement on what constitutes high quality treatment for alcohol use disorder (AUD). National and international clinical guidelines recommend the provision of evidence–based psychological treatment such as motivational interviewing (MI) and cognitive behavioral therapy (CBT) [1–3]. However, few people with AUD seek help for their problems and those who do tend not to seek treatment until several years after developing the disorder [4, 5]. Additionally, international studies show that people who seek help to curb their alcohol problem prefer treatment to be conducted outside conventional health care settings and opening hours [6, 7]. This is largely due to the shame and stigma attached to AUD, but also people may wish to deal with their problems on their own, lack knowledge about existing treatment options, or have poor access to treatment during day time because of family obligations, work, or geographic circumstances [8–10]. Since the treatment gap for AUD is considerable and there is evidence that compliance with AUD treatment is low, with high numbers of no shows and early dropouts [11, 12], this highlights the need for innovation in treatment delivery.

Introducing internet-based psychological treatment may be one way to minimize barriers to treatment delivery and increase access to evidence-based treatment for individuals with mental health problems like addictive disorders [7, 13, 14]. The potential benefits include ease of access (accessible 24/7 from different locations) and capacity to reach a wide range of users in a cost-efficient manner (due to less face-to-face time with the therapist) [15]. Internet-based psychological treatment is typically delivered as an interactive self-help program, comprising components such as symptom questionnaires, daily behavior monitoring with automated feedback, multimedia content, and exercises allowing users to practice coping skills [16, 17]. It can be delivered over a set time period as either 1) unguided Internet-based treatment: pure self-help programs that are not guided by a therapist; 2) guided internet-based treatment: more intensive programs that are guided online by a therapist; 3) blended treatment: consisting of online therapist-guided modules combined with face-to-face sessions.

Unguided internet-based self-help programs are considered useful for getting in contact with drinkers who avoid seeking traditional treatment due to fear of stigma, lack of knowledge about existing treatment options, or ambivalence about whether treatment is needed [7, 9], and such programs may lead to reduction in alcohol consumption in non-treatment-seeking/sub-clinical AUD samples [18–21]. However, although such programs are flexible and easy to access and use, non-compliance is a major challenge [18]. Hence, this type of intervention may primarily offer patients with AUD the opportunity to assess their drinking and degree of functionality, operating as a discrete information tool rather than as a treatment strategy per se. Guided internet-based programs, on the other hand, involve a certain level of contact with a therapist, typically in the form of text messages or emails. Such interventions may function as important and effective treatment strategies [22, 23]. Qualitative studies show that personal feedback and support are perceived positively by psychiatric patients, allowing them to use the program optimally and keeping them motivated [24, 25]. Further, several systematic reviews on the use of internet-based treatment for treating common mental health disorders have found that offering personal support and guidance during online treatment improves clinical outcomes and is associated with higher levels of treatment completion [23, 26, 27]. In addition, a large meta-analysis based on patient data from AUD treatment reported that guided internet-based treatment showed significantly better outcomes than unguided internet-based treatment [23].

A very recent study showed that unguided treatment may, in some cases, have a similar effect as guided treatment [28], but, overall, guided Internet-based treatment is the most promising.

A more novel approach, blended treatment, combines internet-based and face-to-face treatments into one integrated protocol [29–31]. Using this approach, part of the face-to face treatment is replaced by internet components, while the traditional face-to-face relationship between the therapist and patient is retained, thus adding an extra dimension to guided internet-based treatment. Including the face-to-face element ensures that patients benefit from a supportive therapeutic relationship, which is likely to increase motivation to complete treatment [32]. The Internet-based element, however, provides flexibility, allowing patients to access the treatment modules at the time and place of their choosing. Through an internet-based platform, therapists can give continuous feedback to patients and help them to stay on track with treatment [32, 33]. By extending access to treatment in terms of providing online sessions, the number of required face-to-face sessions can potentially be reduced, which may result in cost-savings [34]. However, studies are warranted addressing the clinical and cost-effectiveness of blended treatment.

Mental health care institutions are increasingly introducing blended treatment, and an evidence base has emerged demonstrating the clinical and cost-effectiveness of this type of treatment for a number of psychiatric disorders [14, 29, 31, 35–39]. Studies on the development and implementation of blended treatment for substance use disorders are still scarce [40]. In the context of AUD treatment, no studies have, to our knowledge, compared the effects of blended and traditional face-to-face treatments on a larger scale. This is despite the fact that many AUD patients, particularly those with more severe AUD, may benefit from combining face-to-face sessions with internet modules within the same treatment protocol [7, 41–43], thereby still involving face-to-face contact and making treatment easier to complete. Notwithstanding, blended treatment targeting AUD is currently offered at the Jellinek outpatient institutions in the Netherlands. The Jellinek blended treatment protocol is highly structured and based on evidence-based psychological treatment consisting of MI and CBT [1, 3], and both the clinical and implementation experiences have been positive. Further, a pilot-study testing this treatment program in three outpatient institutions in Denmark has shown promising preliminary results [44]. Therefore, the present large-scale study, “BLEND-A: Blending internet treatment into conventional face-to-face treatment for AUD”, was designed and developed.

The central aim of the BLEND-A Study is to examine and evaluate the effectiveness of the blended treatment program in an implementation framework allowing a country-wide upscale of AUD-treatment reach. We hypothesize that:

1. BLEND-A (internet-based modules combined with face-to-face outpatient treatment within the same protocol) will lead to a 10-percentage point better treatment compliance (primary outcome), measured as decreased premature dropout from treatment (dropout from treatment before planned conclusion), compared to TAU (face-to face outpatient treatment).
2. BLEND-A will be more effective in reducing alcohol consumption (secondary outcome), measured via self-report 6 months after treatment intake, compared to TAU
3. BLEND-A will lead to better quality of life (secondary outcome) 6 months after treatment start compared to TAU.
4. BLEND-A will be a cost-effective treatment approach, comparable to face-to-face treatment (secondary outcome).

## Methods

The study was retrospectively registered on the 1st of September, 2020 in Clinicaltrials.gov (NCT04535258). The reporting of this study is in accordance with the Standard Protocol Items: Recommendations for Interventional Trials (SPIRIT) guidelines [45, 46].

### Study design

The study is a pragmatic, stepped wedge cluster randomized controlled trial [46].

After implementation of the BLEND-A protocol in the participating treatment institutions, we expect that 25–40% of the consecutively enrolled patients will be offered blended treatment and accept to receive it. The decision of whether a patient will receive blended treatment will be made by the therapist and patient together during the first treatment session, taking into consideration the participant’s education, literacy, and computer skills.

Patients not receiving blended treatment are expected to continue receiving face-to-face treatment only. Up to 40 % of the patients are assumed to make use of the possibility of blended treatment. Thus, randomization to BLEND-A treatment and face-to-face treatment is not considered possible at the individual level due to the element of shared decision making in the blended care protocol. Instead, we will use a stepped wedge cluster randomized design [47] and randomize the treatment institutions to implement a new routine in which BLEND-A is offered to patients as a possible alternative to face-to-face treatment.

The BLEND-A study design is, thus, a pragmatic, stepped wedge cluster randomized controlled trial of a blended treatment alcohol intervention incorporating evaluations of the clinical effectiveness of the intervention, its economic impact, its perspectives for sustainable implementation in routine practice, and its impact on patient wellbeing and functioning.

### Study procedure and randomization

All 61 publicly funded Danish treatment institutions were invited to participate in the effectiveness and implementation study. A total of 18 treatment institutions are included in the study. The included institutions are scattered all over the country, and the catchment areas cover approximately 30% of the Danish population.

**Fig. 1:**
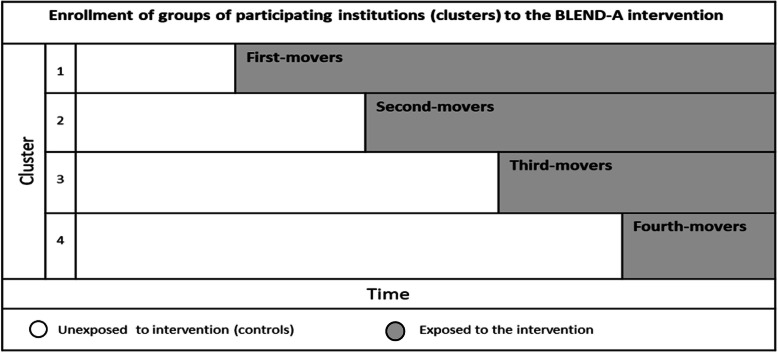
Enrollment of groups of participating institutions (clusters) to the BLEND-A intervantion

The stepped wedge cluster randomized design includes an initial period in which no clusters are exposed to the intervention. Subsequently, at regular intervals of 3 months, one cluster or a group of clusters are randomized to cross from the control group to the intervention group. In the present study, the participating institutions were randomized into 1) first-movers, 2) second-movers, 3) third-movers, and 4) fourth-movers (see Fig. 1.).

First-movers will implement BLEND-A first, and second-movers will implement BLEND-A 3 months later. Third-movers and fourth-movers will implement BLEND-A after another 3 and 6 months. Hence, the patients being treated at an institution before implementation will serve as control groups until the institution implements BLEND-A.

The randomization of the institutions into the four waves of movers occurred by 1) attributing the participating institutions a random number, and 2) elaborating six different models for implementing the institutions (numbers) into the different waves of the study. The randomization procedure was designed by the research group. The models were placed in separate envelopes and then an independent staff member (a statistician not part of the research group) selected one, which was subsequently applied to designate the institutions into first-, second-, third- or fourth movers.

All individuals seeking treatment for AUD at the participating treatment institutions during the study period will be invited to participate as a routine part of the intake procedure at the institutions.

### Interventions

#### Treatment as usual

In Denmark, the public treatment for AUD is delivered by the local governments in the municipalities, and the treatment is free for patients. No referral is needed for treatment [48]. By law, the local governments can choose to establish their own treatment institutions or ask an organization to deliver the treatment. All the treatment institutions in the present study are operated by the municipalities. At the clinics, patients´ psychological treatment consists of MI and CBT [48–50]. Pharmacological treatment may also be provided, administered by the clinic or in collaboration with the patients’ General Practitioners. The psychosocial interventions are typically delivered during individual weekly or bi-weekly sessions alone or in combination with group therapy, and the duration typically varies from 4 to 6 months. The staff typically includes nurses, social workers, therapists, psychologists, and medical doctors, all of whom have received post-graduate training in the treatment of addictions [48].

#### BLEND-A treatment

As preparation for the study, the blended treatment approach platform from the Jellinek clinics was translated into Danish by the study group in the spring of 2017. Therapists and patients from three alcohol treatment institutions (Svendborg, Haderslev, and Kolding) in Denmark were involved in translating and adapting the treatment protocol. The internet-based modules as well as the protocol for the face-to-face sessions in the treatment pathway were translated, adjusted to Danish culture, and adapted accordingly.

The fully translated treatment protocol, internet-based modules, and procedures for access and data flow, were pilot tested in 2018 to explore usability, acceptability, technical and organizational integration, adaption to Danish context, and preliminary effectiveness of the intervention. Two therapists from each of the participating alcohol treatment institutions (Svendborg, Haderslev, and Kolding) implemented the pilot BLEND-A in a total of 22 individual treatment pathways. The pilot test ran for 6 months. After the pilot-study, focus group interviews were conducted with patients and therapists [44]. The treatment protocol was adjusted during the testing process.

The Blend-A platform is hosted by the Dutch company, Minddistrict, and it is accessible via any given web-browser. The online sessions follow the course of the ordinary treatment offer; a fixed structure starting with therapy information, followed by multiple exercises and homework assignments, training in optional skills adjusted to the patients’ individual needs, and relapse prevention. The platform offers four modules. The first module, Welcome and preparation, lasts for approximately 30 min. The second module comprises nine submodules addressing different aspects of treatment: a) Motivation, b) Treatment goals, c) Risk situations, d) Functional analysis, e) Relapse prevention strategies, f) Coping with craving, g) Restructuring of thoughts, h) Communication training focusing on saying no to alcohol, and i) Evaluation of the treatment course (each lasting approximately 30 min). The third module comprises seven submodules that are optional: a) Social skills - small talk, b) Social skills – tackling criticism, c) Social skills – giving criticism, d) Mood management, e) Stress management, f) Problem solving strategies, and g) Tackling relapse (each lasting approximately 30 min). The fourth module includes six submodules focusing on aftercare, primarily monitoring alcohol use and quality of life (each lasting approximately 15 min).

The therapist can gradually add online sessions to the patient’s individual platform. The online sessions contain text and videos with information as well as assignments. Patients receive online feedback about assignments from their therapist. The platform allows the sharing of information and homework assignments with significant others. If a patient stops being active in treatment, the therapist will contact him/her by email, phone, or video, or if needed, invite him/her to resume face-to-face treatment. Thus, the Internet-based modules and face-to-face treatment can be combined in numerous ways.

It is estimated that the active blended treatment course will last for 3 months. Thereafter, the patients can use the platform for relapse prevention for up to 6 months. Upon completion of treatment, the patients will continue to have access to the online treatment platform so they can re-read information and look up exercises.

#### Training of therapists

MI and CBT are the most widely used treatment modalities for AUD in Denmark. Most Danish alcohol therapists have participated in the Basic training for Alcohol Therapists (lasting 16 days), offered by the National Health and Medicine Authority. The training offers a solid introduction to MI and CBT and thereby to the treatment content of the blended protocol. In addition, most treatment institutions offer regular ongoing training in MI and CBT to their therapists.

The BLEND-A Study includes a one-day training session in the BLEND-A protocol for all therapists involved. During the training, they will learn how to navigate the online platform and practice with the different modules. Initially, the training will be provided by experts in the field of internet-based treatment (from the Centre for Telepsychiatry). All therapists from the participating institutions will be offered the training before the implementation phase begins at their institution, and they will receive a manual instructing how to use the platform.

They will thereafter obtain access to the web-based part of BLEND-A via Sundhed.dk and will be encouraged to invite future patients to blended treatment.

#### Intervention fidelity and access to supervision

A key therapist from each of the participating treatment institutions will receive additional training in the protocol and function as a local supervisor. Further, a therapist from each institution will be offered the opportunity to participate in a full day follow-up network meeting every month (10 days per year) during the study period, where the project group will also be represented. In this meeting, practical questions and considerations about how to make the intervention work can be sorted out in order to secure intervention fidelity. In addition, a help desk operated by the Centre for Telepsychiatry will be made available to the key therapists. The use of the internet-based modules will be registered and monitored throughout the study period.

### Data collection

All patients from each of the participating treatment institutions will be followed up in the National Register of Alcohol Treatment (NAB). In accordance with Danish Law, this register contains data on all patients receiving publicly funded treatment for AUD. The register provides data on sociodemographic characteristics, compliance, and alcohol related variables (see Table 1).

**Table 1:** Data collected during the BLEND-A study

Domain	Measure/source	Content	Reference	Time-point
Descriptive variables				
Sociodemographics and treatment data	National Alcohol Treatment Register (NAB)	Gender, age, education, etc. and treatment duration and intensity as well as prior treatment.	[51, 52]	Treatment entry and treatment completion
Primary outcomes				
Compliance	National Alcohol Treatment Register (NAB)	Treatment retention and type of treatment conclusion (i.e. dropout or completion).		Registered on continuous basis in the national register
Secondary outcomes				
Alcohol consumption	Alcohol Use Disorders Identification Test (AUDIT)	Items from the AUDIT: Quantitiy/frequency measure of alcohol consumption.	[53]	Treatment entry and 6-month follow-up post treatment
AUD severity	Alcohol Use Disorders Identification Test (AUDIT)	AUDIT asesses AUD, alcohol consumption, drinking behaviors, and alcohol-related problems	[53]	Treatment entry and 6-month follow-up post treatment
	Alcohol Dependence Scale (ADS):	ADS measure the severity of alcohol dependence symptoms.	[54, 55]	Treatment entry and 6-month follow-up post treatment
	Short Inventory of Problems-2 Revision (SIP-2R)	SIP-2R evaluates alcohol-related problems.	[56]	Treatment entry and 6-month follow-up treatment
	National Alcohol Treatment Register/ Addiction severity Index (ASI)	ASI assesses multidimesional addiction severity related to various domains: alcohol and drug use, physical and mental health, employment, legal problems and social functioning.	[51, 52]	Treatment entry and treatment conclusion
Quality of life	European Quality of life - 5 Dimensions (EQ5D)	EQ5D evaluates quality of life on five dimensions: mobility, self-care, usual activities, pain/discomfort, and anxiety/depression.	[57, 58]	Treatment entry and 6-month follow-up post treatment.
Usability of blended treatment	System Usability Scale (SUS)	SUS evaluates the usability of the web-based treatment system.	[59]	6-month follow-up post treatment.

Institutional level data will cover 1 year before treatment start until the end of the study period for all consecutive patients at the participating treatment institutions.

In addition to the register data, all patients seeking treatment at the participating treatment institutions during the study period will be asked to complete a questionnaire at treatment start and after treatment completion 6 months later. The questionnaire will consist of questions on the patients’ contact information, alcohol consumption, consequences of drinking, quality of life and usability of the internet-based platform (see Table 1).

The questionnaire will be administered via tablets at the treatment institutions or by secure mail or telephone interviews conducted by blinded research assistants. If a patient stops treatment before the 6 months follow up, the questionnaire will be emailed to him/her via a safe link or administered over the phone by a researcher. The questionnaire-based data will be collected from patients in both the intervention and the control groups. Data will be collected using REDCap™ (Research Electronic Data Capture), which is a secure web application for building and managing online data entry systems [60], and stored in OPEN, Open Patient data Explorative Network, Odense University Hospital, Odense. Data on use of the BLEND-A modules will be obtained from the platform.

Randomly chosen patients and therapists will be asked to participate in additional qualitative interviews, conducted either individually or in focus groups to investigate how participation in blended care is experienced by the users. An interview guide will be developed, which will allow the patients and therapists to describe both the challenges and opportunities associated with the blended treatment approach [61].

### Data analysis

In a stepped wedge study, the distribution of results across unexposed observation periods is compared with the distribution of results across the exposed observation periods [62]. The data in the present study will be analyzed using an Intention-to-treat approach and by a blinded statistician. In the BLEND-A study, the evaluation will be conducted over a 16-month period during which the proportion of clusters exposed to the blended treatment intervention gradually increases. Adjusting for the systematically different observation periods and for clustering in the data will be accomplished by fitting an appropriate generalized linear mixed model or using generalized estimating equations (GLMM). We may also decide to examine how the impact of implementing blended treatment develops (over time) once it is introduced into a cluster, since the intervention may need an initial period of adjustment before becoming fully embedded in the setting. Hence, the length of the period (up to the current observation) during which the cluster (treatment institution) has been exposed to the intervention may be included in the model as an effect modifier [62].

#### Economic analyses

The economic analysis includes 1) cost comparisons, 2) a cost and benefits comparison, 3) a formal cost-effectiveness incremental cost-effectiveness ratio (ICER: ratio of Net Costs to Net quality-adjusted life-years (QALYs)), and 4) a business case analysis. Cost comparisons entail cost estimation of either of the interventions (BLEND-A or TAU), including costs that are directly related to the interventions and costs associated with the impact of the interventions on patients, i.e. health care utilization, social care, patients’ own resources and labor supply/productivity. Data on the use of resources will be extracted from population registries maintained by Statistics Denmark and The Danish Health Data Authority, and will be supplemented with data from the municipalities’ own databases and ad hoc measurements (especially for resources directly related to the interventions). Net costs, ∆C = (Costs(BLEND-A) minus Costs(TAU)), will be assessed with means, medians, and statistical tests, and decomposed into health net costs and social net costs. Prices and wage rates will be based on negotiated/market prices, DRG-rates and similar. Sub-group analyses will be performed for different patient categories.

Costs and benefits (premature dropouts, decreased alcohol intake, and increased quality of life at 6 months after treatment intake) in the two intervention groups will be compared and described to assess cost-effectiveness. A formal cost-effectiveness analysis will be performed according to international standards [63–65]. Validity of the EQ-5D, being a generic Quality of life (QoL) instrument, will be empirically assessed in the study by comparing EQ-5D measures with patient reported outcome (PRO) data. ICERs and sensitivity analysis will be estimated with non-parametric bootstrap-based confidence intervals.

Finally, we aim to perform a business case calculation using the Danish framework so that the municipalities can perform their own business cases to assess the consequences of implementing BLEND-A.

#### Power calculations and number of participants needed in the study

In 2014, 31% of all patients seeking treatment at a publicly funded alcohol treatment institution completed the treatment pathway as planned [12, 66]. We hypothesize that implementing BLEND-A will lead to a 10-percentage point higher level of compliance compared to TAU, i.e. increasing the total number of patients who complete the treatment course at the treatment institution according to the NAB register.

We wish to be able to identify a 10-percentage point increase in overall treatment completion (from 31% now with treatment as usual to 41% with BLEND-A) with 80% power and a two-sided significance level of 5%. In order to secure the randomization process, we aim to involve at least two major institutions (enrolling more than 200 new patients per year, typically 300–600 patients), and additionally 6 or more minor treatment institutions (typically enrolling 60–80 new patients per year [66]). The power calculation, based on Hemming et al.’s recommendations for stepped wedge cluster randomized trials [47, 67], estimates that a total of 1800 individuals, enrolled in treatment at such 8 treatment centers and enrolled in four steps (the first two steps were 6 months apart, the others steps were 4.5 months apart) during a period of 20 months, will be sufficient to detect a 10 percentage point improvement with at least 80% power. Register data stemming from the year prior to the start of enrollment will be regarded as the first step. The figure below shows the power as a function of the improvement for various situations of intra-center-correlations, and the power is exceeded when the effect is a 10-percentage point improvement from 31 to 41% as specified.

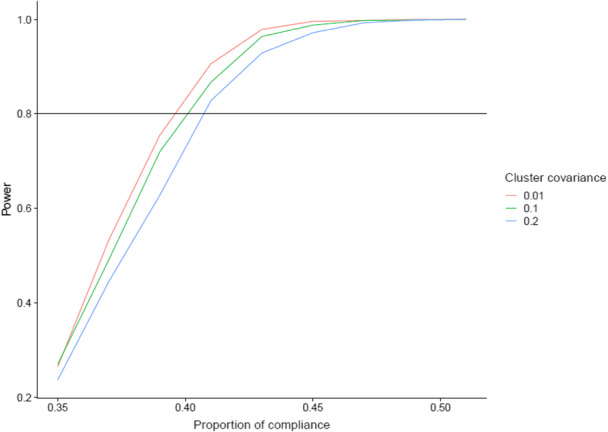



### Ethical considerations

Since blended treatment for AUD has already been implemented in other countries, the ethical problems are scarce. Furthermore, although BLEND-A will be offered to all patients at the participating institutions, we expect that the intervention will only be implemented in 25–40% of the treatment pathways, and, thus BLEND-A will not be the only choice offered to the patients, but rather another option for treatment delivery.

The BLEND-A study has been approved by The Regional Committees on Health Research Ethics for Southern Denmark (S-201901166G). The ethics approval covers all sites involved in the study. All participants will provide written informed consent before taking part in the study.

## Discussion

The main purpose of the BLEND-A study is to examine compliance with and the effectiveness (including cost-effectiveness) of an evidence-based blended treatment program to be used in Danish alcohol treatment institutions. Specifically, BLEND-A will implement the translated and adapted treatment protocol, developed in the BLEND-A pilot study. Technically, the BLEND-A study will employ a secure internet-based treatment platform and provide access to the blended treatment program through the Danish e-Health Portal: Sundhed.dk, which allows access for citizens and health professionals. This approach will enable wide dissemination since all public health care providers are already able to connect to the portal. The combination of a national portal and a scalable and proven software solution will enable the project to take up new partners as they join the program at low cost. The cost will be kept low at the local level as IT investments are limited to training in the software and treatment workflows in the blended treatment approach.

Most of the online psychological treatments developed to target problematic alcohol use have so far been brief, low-intensity, automated programs delivered without guidance from a therapist [14, 18, 68, 69]. The problem with unguided internet-based programs is that uptake and compliance is low and translation of therapy content into daily life is difficult [70, 71]. Further, even with therapist guided treatment, compliance and treatment adherence may be lower than for face-to-face treatment [72]. While unguided interventions may be optimal for cases not fulfilling diagnostic criteria for AUD or mild AUD cases, the blended approach could be more feasible and easier to implement among patients with moderate and even severe AUD, since it still includes face-to-face treatment [7, 41–43].

When implementing Internet interventions, reluctance among patients, clinicians, and other stakeholders represent an important barrier [43, 73]; however, blended treatment seems to reduce this barrier to a considerable extent. In a stakeholder survey conducted in eight European countries involving 175 mental health organizations, results revealed greater acceptability of blended treatment compared to stand­alone internet treatments: for mild mental disorders, 47% would recommend internet-based treatment only and 70% blended treatment, but for moderate mental disorders the corresponding figures were 15.7 and 57.2%, representing a marked difference. The same discrepancy was found for severe mental disorders, with 1.9% recommending internet-based treatment and 27% blended treatments [43]. Thus, stakeholders seem much more inclined to implement blended treatment for moderate and even more severe mental disorders than they are to implement unguided and guided standalone interventions.

These findings are in line with the BLEND-A pilot study involving therapists and patients from three treatment institutions. A clear advantage of blended treatment was that the therapist and the patient could decide together whether to continue with blended treatment or proceed with TAU. Further, the therapist could assess and refer the most severe and complex AUD patients to detoxification and inpatient treatments. The therapists participating in the BLEND-A pilot study found that the blended-care approach could easily be integrated in outpatient community-based treatment for AUD. They did not consider giving written feedback on the patients’ internet-based homework as being difficult and they perceived that they had more time to reflect and focus than when giving feedback in a direct conversation. The patients participating in the pilot study expressed that they liked the mixture of face-to-face therapy and internet-based homework. They found it helpful that blended care involved homework assignments, including reading and writing tasks, and that it was possible to repeat tasks if they felt that they needed a brush-up. Finally, the pilot study indicated that a new group of patients may be reached by the blended care treatment offer. Several patients approached the treatment center specifically because they had learned about the possibility of receiving treatment over the internet that is still guided by professional therapists. These patients claimed that they would not have sought treatment if it had involved face-to-face treatment only. They appreciated the flexibility and the discretion that blended care offered [44].

The improved access to flexible treatment due to the online part of blended treatment may also prevent premature treatment drop-out, which is obviously an advantage. It has, however, also been suggested that face-to-face treatment can be too long [50]. If the treatment pathway and thereby the interaction with the therapist lasts too long, the patient may not experience an optimal transfer of new competences into daily life and the therapy may turn into a less structured and more private kind of conversation [74–76]. Blending face-to-face sessions with internet-based modules may not only be a way of preventing early treatment drop-out due to ease of access but also of preventing the patient from becoming dependent on the therapist due to patient empowerment [75, 76]. Additionally, blended treatment may help to prevent ‘therapist drift’ since the internet modules and exercises not only provide a clear working structure for the patient but also for the therapist [77]; the therapist is nudged to incorporate the full therapy protocol in the sessions, thus improving compliance with the therapy protocol on the therapist’s side [78].

Blended treatment is expected to improve compliance with treatment and prevent early drop-out. Furthermore, the overall effect of treatment for AUD might increase since more patients finalize treatment. Underserved people suffering from AUD will thus be offered state-of-the-art, evidence-based, blended treatment, and the reach of existing alcohol treatment providers will be extended. By enlarging the reach of alcohol treatment services, more people can benefit from them, including those who otherwise are not able to access services due to geographical distances, study and work schedules, perceived stigma attached to seeking treatment, and other inequitable circumstances.

The BLEND-A study will be performed and implemented in operating treatment institutions; thus, it may increase the likelihood that knowledge and experience gained from performing the study will stay in the treatment institutions after study conclusion. After the study, the treatment protocol will be offered to the treatment institutions for free, and this may help bridge the gap between the need for treatment and its provision.

Implementing and evaluating BLEND-A in routine care is the first step to creating a solid basis for integrating internet-based modules in treatment for AUD, and in particular forms the basis for estimating cost-effectiveness of the intervention. Allowing for part of the treatment to be performed via the internet with face-to-face therapist support also opens the possibility for adapting the treatment to comorbid mental disorders the patient may be suffering from. It also offers the opportunity for continuing treatment during hospitalization and during aftercare.

## Data Availability

The dataset is available on request from the authors, provided this is compliant with the national legislation and with the decisions of the ethical committee.

## Abbreviations

ADS: Alcohol dependence scale
ASI: Addiction severity Index
AUD: Alcohol use disorder
AUDIT: Alcohol use disorders identification test
CBT: Cognitive behavioral therapy
EQ5D: European quality of life - 5 dimensions
GLMM: Generalized estimating equations
ICERs: Incremental cost-effectiveness ratio
MI: Motivational interviewing
OPEN: Open patient data explorative network
QALYs: Quality-adjusted life-years
QoL: Quality of life
REDCap: Research electronic data capture
SIP-2R: Short inventory of problems-2 revision
SUS: System usability scale
TAU: Treatment as usual
